# Deep learning-based pathological prediction of lymph node metastasis for patient with renal cell carcinoma from primary whole slide images

**DOI:** 10.1186/s12967-024-05382-6

**Published:** 2024-06-14

**Authors:** Feng Gao, Liren Jiang, Tuanjie Guo, Jun Lin, Weiqing Xu, Lin Yuan, Yaqin Han, Jiji Yang, Qi Pan, Enhui Chen, Ning Zhang, Siteng Chen, Xiang Wang

**Affiliations:** 1 Pathology Center, Shanghai General Hospital, Shanghai Jiao Tong University School of Medicine, Shanghai, China; 2 Department of Urology, Shanghai General Hospital, Shanghai Jiao Tong University School of Medicine, Shanghai, China; 3 Department of Pathology, Dongtai People’s Hospital, Dongtai, Jiangsu China; 4 Department of Urology, Ruijin Hospital, Shanghai Jiao Tong University School of Medicine, Shanghai, China; 5 Department of Urology, Renji Hospital, Shanghai Jiao Tong University School of Medicine, Shanghai, China

**Keywords:** Renal cell carcinoma, Lymph node metastasis, Whole slide images, Deep learning, Prognosis

## Abstract

**Background:**

Metastasis renal cell carcinoma (RCC) patients have extremely high mortality rate. A predictive model for RCC micrometastasis based on pathomics could be beneficial for clinicians to make treatment decisions.

**Methods:**

A total of 895 formalin-fixed and paraffin-embedded whole slide images (WSIs) derived from three cohorts, including Shanghai General Hospital (SGH), Clinical Proteomic Tumor Analysis Consortium (CPTAC) and Cancer Genome Atlas (TCGA) cohorts, and another 588 frozen section WSIs from TCGA dataset were involved in the study. The deep learning-based strategy for predicting lymphatic metastasis was developed based on WSIs through clustering-constrained-attention multiple-instance learning method and verified among the three cohorts. The performance of the model was further verified in frozen-pathological sections. In addition, the model was also tested the prognosis prediction of patients with RCC in multi-source patient cohorts.

**Results:**

The AUC of the lymphatic metastasis prediction performance was 0.836, 0.865 and 0.812 in TCGA, SGH and CPTAC cohorts, respectively. The performance on frozen section WSIs was with the AUC of 0.801. Patients with high deep learning-based prediction of lymph node metastasis values showed worse prognosis.

**Conclusions:**

In this study, we developed and verified a deep learning-based strategy for predicting lymphatic metastasis from primary RCC WSIs, which could be applied in frozen-pathological sections and act as a prognostic factor for RCC to distinguished patients with worse survival outcomes.

**Supplementary Information:**

The online version contains supplementary material available at 10.1186/s12967-024-05382-6.

## Background

Renal cell carcinoma (RCC) is a highly prevalent cancer, with the sixth incidence rate in male malignancies and the ninth incidence rate in female malignancies [1–3]. In 2021, RCC patients accounted for around 3–5% of new estimated cancer patients [1, 4]. RCC has multiple pathological subtypes, including clear cell RCC (ccRCC), papillary RCC (pRCC), chromophobe RCC (ChRCC) and other rare types [5]. Among all the histological subtypes, ccRCC is the predominant subtype and comprises up to 70–80% of all RCC cases [6]. The primary treatment of localized RCC is nephrectomy or ablation [7, 8]. Patients with localized RCC usually have acceptable clinical outcomes after treatment [9]. However, metastasis or recurrence RCC patients have extremely high mortality rate, with the 5-year overall survival around 8-11.7% [6, 10, 11]. It is estimated that around 30% of localized RCC patients eventually progress to metastasis even after treatment [7].

Lymph node involvement represents for regional spread of RCC, which may proceed to distant metastasis eventually [7, 12]. It is reported that RCC patients with lymph node involvement have worse clinical outcomes with a median recurrence-free survival of 4 months [12, 13]. However, the benefit of lymph node dissection for RCC patients remains controversial [12]. In addition, some micrometastasis in lymph nodes may be undetected by pathologists, which needs serial sections of lymph node histological slides [14]. It is necessary that an optimal criterion for lymph node dissection and serial sections of histological slides.

Intriguingly, with the further application of artificial intelligence on medical sciences, there appear some studies on prediction of lymph node involvement based on histological features [15–17]. These studies were targeted on prostate cancer, colorectal cancer and gastric cancer, through deep learning techniques [15–17]. In this study, we are the first to utilize deep learning method to predict the lymph node involvement based on whole slide image (WSI) of RCC.

Here, in this study, we developed a deep learning-based strategy for predicting lymphatic metastasis from primary WSI. We further verified the deep learning-based prediction of lymph node metastasis (D_LNM_) model in frozen-pathological sections and the potential clinical use of prognosis prediction of patients with RCC in multi-source patient cohorts.

## Materials and methods

### Data sources

Our study recruited three large patient-based cohorts from Shanghai General Hospital (SGH), Clinical Proteomic Tumor Analysis Consortium (CPTAC) [18, 19], and the Cancer Genome Atlas (TCGA) [18]. All the included patients shall have pathological diagnosis of RCC. Additional inclusion criteria for this study included: (i) with complete clinicopathological information and disease-free survival follow-up information; (ii) without severe surgical complications and other types of malignant tumors; (iii) without postoperative drug therapy (iv) with access to hematoxylin-eosin stained (H&E) slides or WSI. Basic clinical characteristics of patients from three independent patient cohorts were shown in Table S1. The TCGA cohort was randomly divided at the patient level in training set (80%) and testing set (20%) for the training and internal verification of the deep learning-based model. The SGH cohort and CPTAC cohort were used as the independent external verification cohorts.

### SGH cohort

The SGH cohort recruited 486 patients who underwent partial or radical nephrectomy operative treatments and were pathologically diagnosed as RCC from January 2012 to September 2019 in SGH. After excluding the participants failed to meet the inclusion criteria, 402 cases were suitable for this study, including 307 ccRCC case, 51 pRCC cases, and 44 ChRCC cases. The corresponding H&E-stained slides of formalin-fixed and parrffin-embedded sections were retrieved from the pathology database in SGH, which were further scanned at SGH with Leica Aperio AT2 scanners at 200× equivalent magnification for the digitalization of slides.

### TCGA cohort

A total of 381 patients from the TCGA database were also included in the study, which contained diagnostic pathological images met with the inclusion criteria mentioned above. The corresponding H&E-stained images with 200× equivalent magnifications were further acquired from the same database, including 307 ccRCC case, 51 pRCC cases, and 44 ChRCC cases.

### CPTAC cohort

In addition, 112 cases with ccRCC from the CPTAC cohort with digitized WSIs, which were met with the inclusion criteria mentioned above, were also included. The CPTAC cohort was used as an external validation cohort to estimate the generalization performance of the D_LNM_ model.

### Image pre-processing

All available WSIs were strictly reviewed by two experienced pathologists to ensure that each slide had representative tumor regions. Since the WSIs were labeled in slide-level without manual annotations of tumor regions, we firstly segmented the WSI to remove non-tissue regions and exclude any holes. During the tessellation process, we segmented the whole slide within the segmented foreground contours into 256 × 256 patches at 100× magnification without overlap. All the patches extracted from the same slide were then identified as the instance of the WSI. Image patches and their coordinates were then stored as hdf5 hierarchical format [20].

### Deep learning strategy based on multiple instance learning

**Fig. 1:**
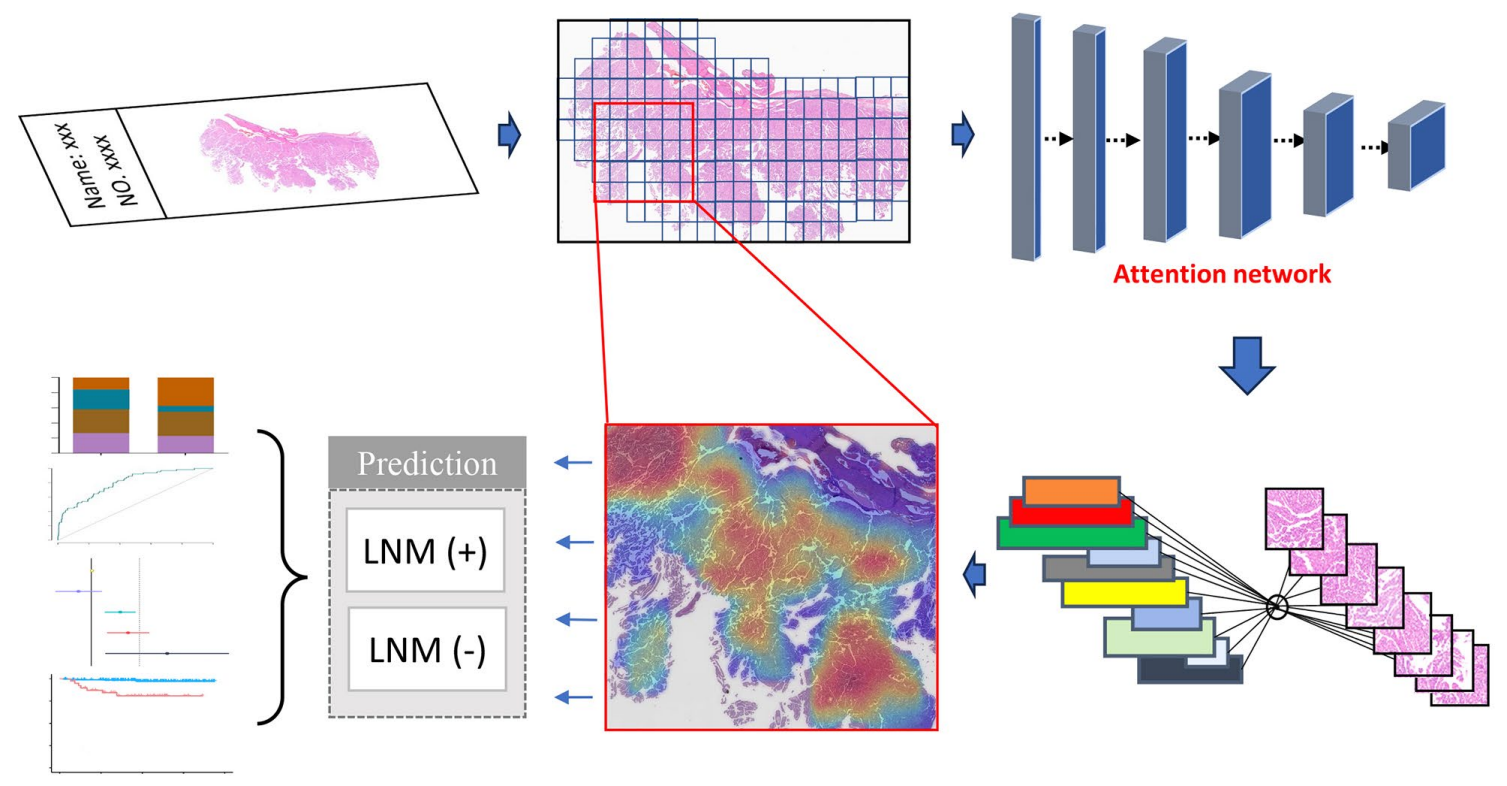
Architecture of the deep neural network in this study

In this study, we applied a clustering-constrained-attention multiple-instance learning method [20] to accurately perform instance-level clustering without any manual annotations. The overall architecture of the hybrid neural network was displayed in Fig. 1. Based on the patches of WSI, the multiple-instance learning strategy achieved the classification of WSI in view of the entire information from the slide. Firstly, we carried out dimensionality reduction from raw image data through encoding each 256 × 256 patch into a descriptive 1024-dimensional feature vector using a ResNet50-based CNN with fixed parameters pretrained on ImageNet32 [21–23]. Feature information from the whole region of each WSI was then assembled through attention-based pooling and contributed to predict the classification of a slide [20, 24]. Two fully-connected layers were set followed by the activation function of rectified linear unit (ReLU). Referring to the predicted attention weight of each patch, the attention pooling would average the representative features of a slide for prediction. We implied cross-entropy loss function for multiple tasks to predict tumor group.

### Evaluation of the deep learning-based model

In this study, evaluation of the D_LNM_ was performed through receiver operating characteristic curve (ROC) analysis with area under curve (AUC). The best cut-off value for the prediction model was calculated through ROC analysis, with the best specificity and sensitivity. Significant differences of AUC values were evaluated through DeLong methods [25]. Survival analysis was performed via Kaplan–Meier (KM) curve with hazard ratio (HR) and 95% confidence interval (CI) to compare different overall survival (OS) outcomes.

## Results

### Overall performance of the D_LNM_

**Fig. 2:**
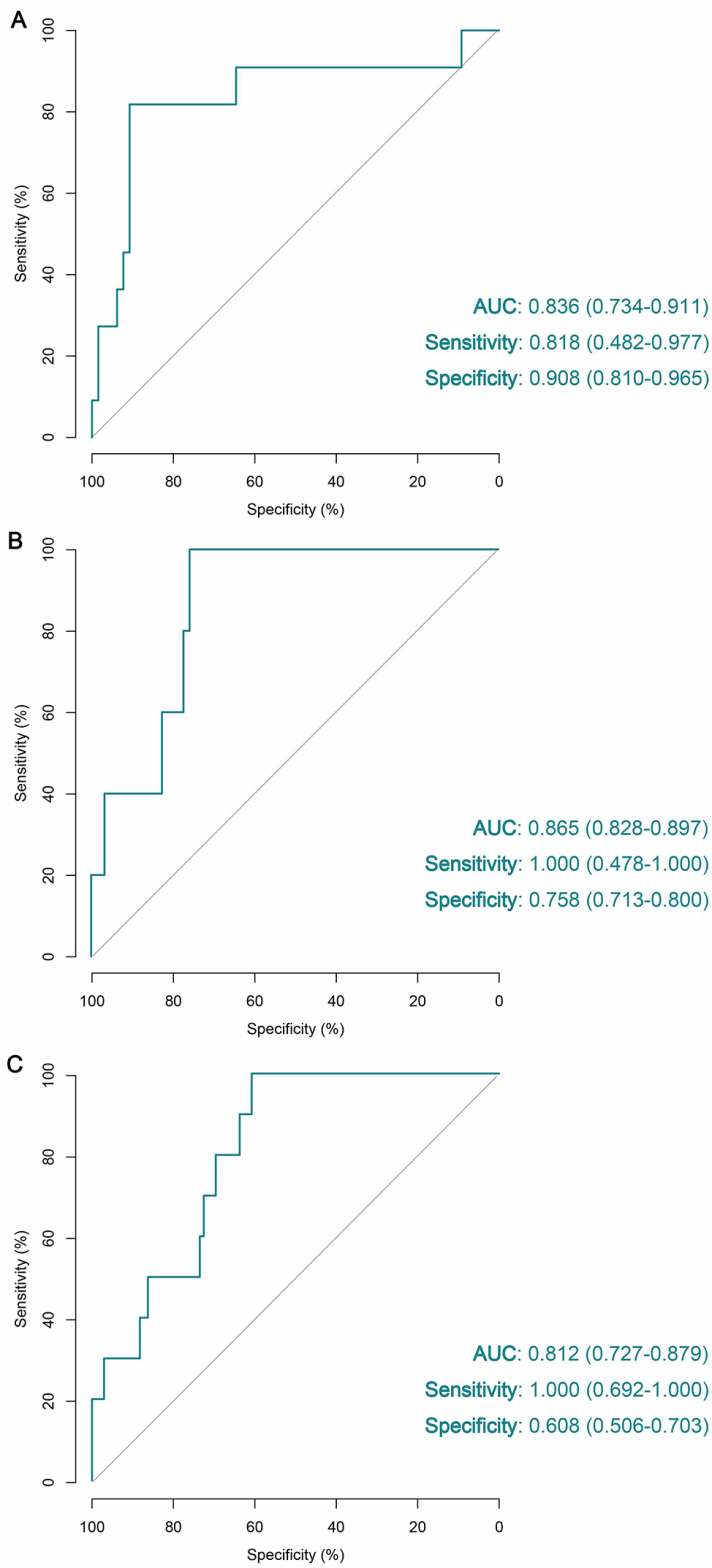
Overall performance of the D_LNM_ in the internal validation and external validations through receiver operating characteristic curve. (**A**) Evaluation of the D_LNM_ in the TCGA testing cohort. (**B**) Verification of the D_LNM_ in the SGH cohort. (**C**) Verification of the D_LNM_ in the CPTAC cohort. D_LNM_, deep learning-based prediction of lymph node metastasis; TCGA, the Cancer Genome Atlas; Shanghai General Hospital, SGH; CPTAC, Clinical Proteomic Tumor Analysis Consortium; AUC, area under the curve with 95% confidence interval

Based on the TCGA cohort, we explored the slide-level classification performance of the deep neural network in the task of detecting lymph node metastasis (LNM) from primary tumor images. The TCGA cohort was firstly randomly partitioned into a training set (80% of cases) and a testing set (20% of cases), stratified by each class. We adopted any level of LNM as positive reference standard in the training. All models are trained for at least 50 epochs according to the monitored validation loss of each epoch [20]. The D_LNM_ was then developed based on the optimization model with the lowest validation loss. Evaluated by the ROC analysis, our D_LNM_ achieved an AUC of 0.836 (95% CI 0.734–0.911) in the internal testing set (Fig. 2A). When the cut-off value was set as 0.382, the _DLNM_ achieved the best general performance with a sensitivity and specificity of 0.818 (0.482–0.977) and 0.908 (0.810–0.965), respectively.

### Validation on the external cohort

The robustness of a prediction model might be influenced by different datasets due to the data-specific variables among different patient cohorts, which arouses the importance for validating models in external patient cohorts [11]. Therefore, we recruited external validations in SGH cohort and CPTAC cohort to further evaluate the generalization performance of our D_LNM_. As shown in the Fig. 2B and C, the D_LNM_ also performed well in external validations, with AUC of 0.865 (0.828–0.897) and 0.812 (0.727–0.879) in the SGH cohort and CPTAC cohort, respectively, which suggested the good generalization of the D_LNM_.

### Application of D_LNM_ in frozen-pathological sections

**Fig. 3:**
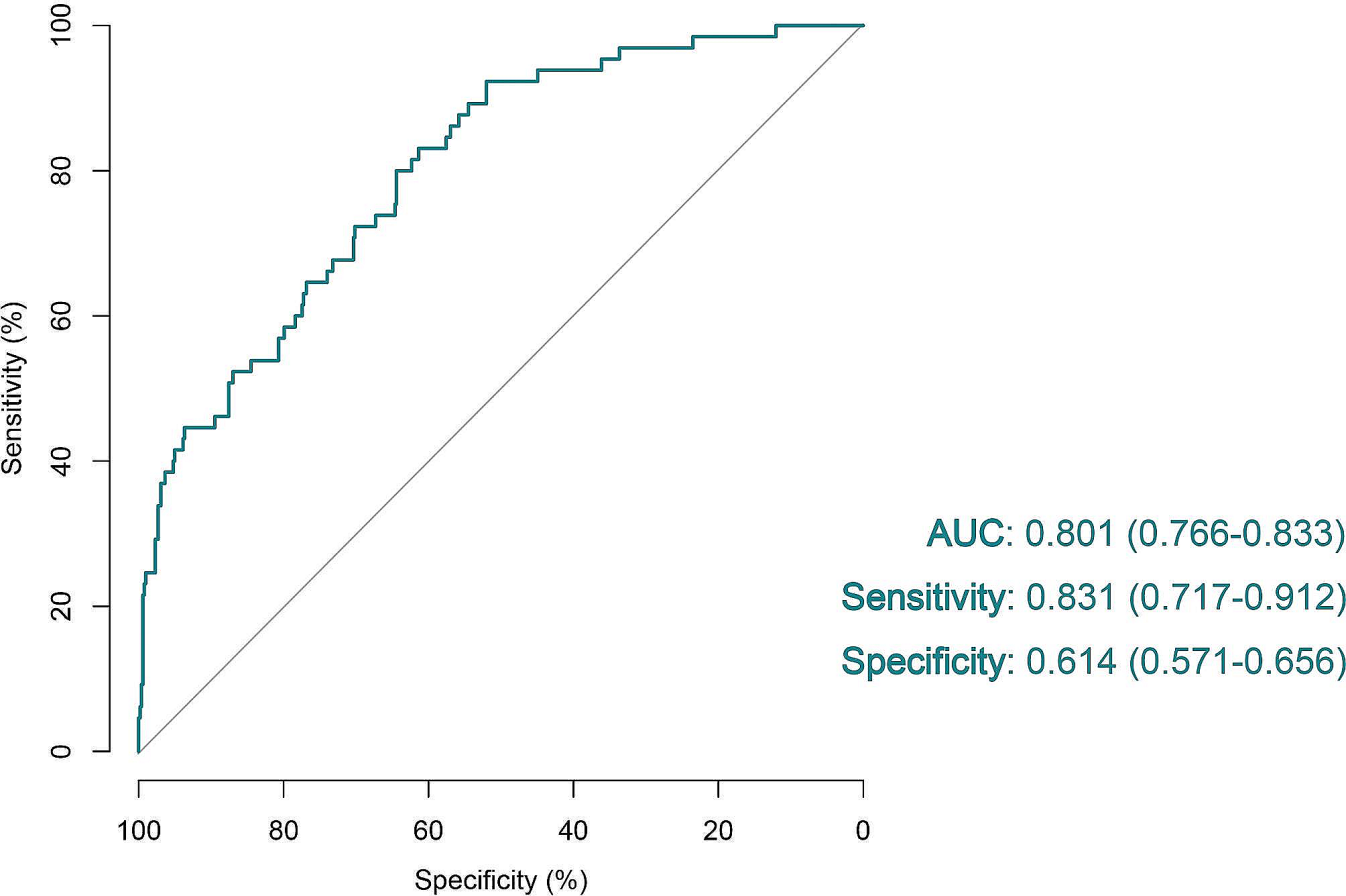
Performance of the D_LNM_ in frozen sections of renal cell carcinoma. D_LNM_, deep learning-based prediction of lymph node metastasis; AUC, area under the curve with 95% confidence interval

For localized RCC, surgery is the only curative treatment with high-quality evidence [26]. For patients with localized disease and clinically enlarged lymph nodes, the lymph node dissection is currently performed for staging purposes. However, as the most common method for preliminary evaluation of LNM before surgery, radiological observation is still restrained by its indirect imaging and low-resolution. Therefore, we further explored whether our D_LNM_ could also be applied in frozen-pathological sections, which could be made in the operations and help to make auxiliary diagnosis during surgery. We retrieved another 588 WSIs from frozen RCC sections in the TCGA dataset and used the analytical framework of the same D_LNM_ without transformation. As shown in Fig. 3, our D_LNM_ could also be applied in frozen-pathological sections, with AUC of 0.801 (0.766–0.833), sensitivity of 0.831 (0.717–0.912) and specificity of 0.614 (0.571–0.656). Our D_LNM_ could accurately and efficiently evaluate LNM status as well as greatly improve the surgery efficiency.

### Prognosis prediction of patients with RCC through D_LNM_

**Fig. 4:**
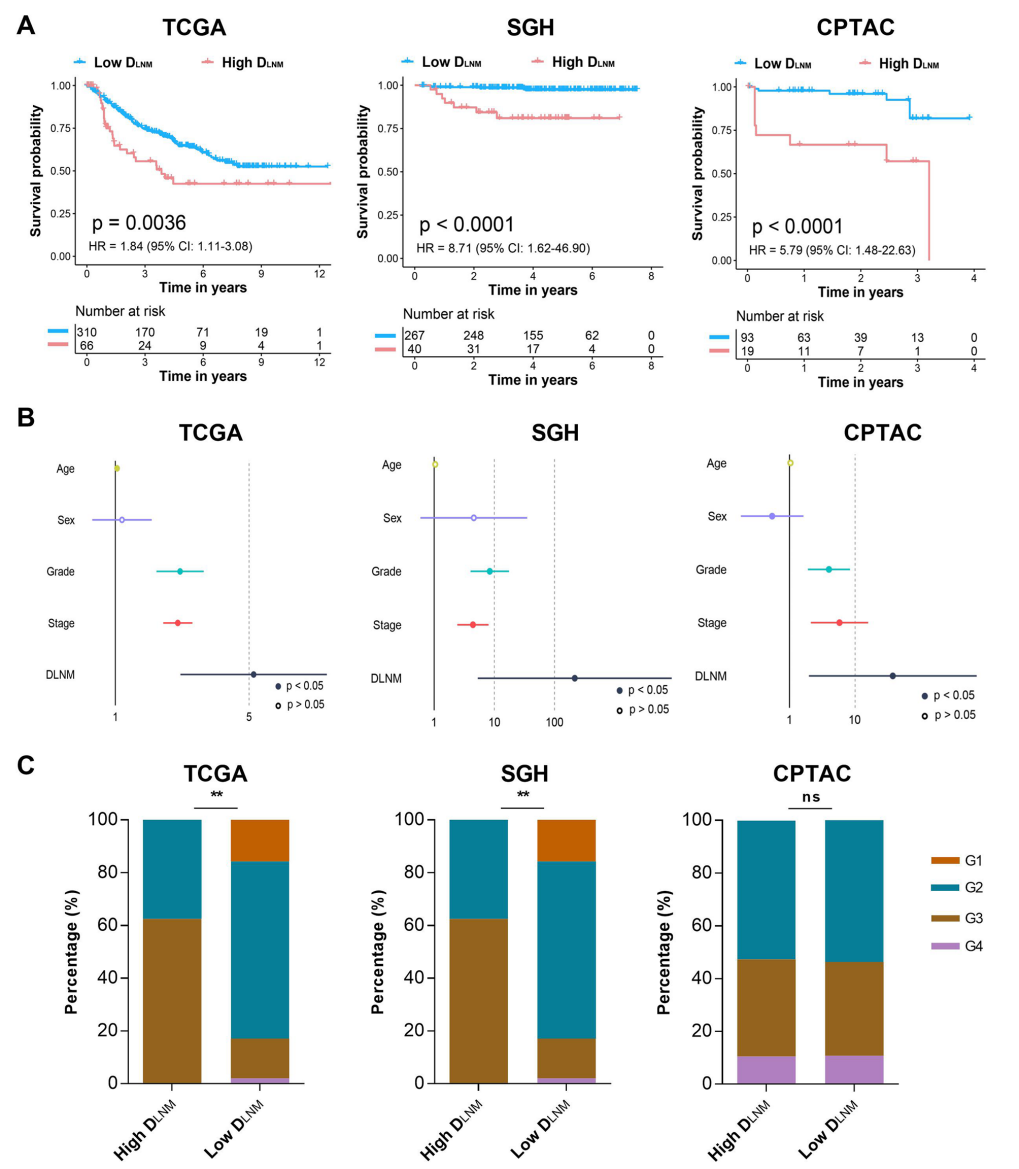
Prognosis prediction of patients with renal cell carcinoma through the D_LNM_. (**A**) Kaplan-Meier survival analysis stratified by D_LNM_ for overall survival in three independent patient cohorts. (**B**) Cox regression analysis of the D_LNM_ for overall survival in three independent patient cohorts. (**C**) Differences in the distribution of renal cell carcinoma with different tumor grades between patients with high or low D_LNM_. D_LNM_, deep learning-based prediction of lymph node metastasis; TCGA, the Cancer Genome Atlas; Shanghai General Hospital, SGH; CPTAC, Clinical Proteomic Tumor Analysis Consortium; HR, hazard ratio; CI, confidence interval; ns, no significant

Prognosis of patients with RCC is influenced by molecular and clinicopathologic factors. Among all risk factors, LNM acts as a key factor in clinical prognosis. Therefore, we further explored whether our D_LNM_ could also provide reliable prognostic information for patients with RCC. Patients were categorized into high-D_LNM_ or low-D_LNM_ groups based on the best cut-off value of our model. KM curve analyses indicated that patients with high-D_LNM_ seemed to have worse survival outcomes compared with patients with low-D_LNM_, with the HR of 1.84 (1.11–3.08, *p* = 0.0036) in the whole TCGA cohort (Fig. 4A). Validations on the external SGH cohort and the CPTAC cohort also confirmed that patients with different predicted D_LNM_ statuses had distinct prognosis during the follow-up, with HR of 8.71 (1.62–46.90, *p* < 0.0001, Fig. 4A) and 5.79 (1.48–22.63, *p* < 0.0001, Fig. 4A), respectively. Further Cox regression analysis revealed that our D_LNM_ could act as a prognostic factor for RCC, illustrating that our D_LNM_ had a promising risk stratification performance in independent patient cohorts (Fig. 4B). In addition, differences in the distribution of RCC with different tumor grades between patients with high or low D_LNM_ were also observed in the study, which revealed that our D_LNM_ might be associated with higher levels of tumor grades (Fig. 4C).

## Discussion

TNM classification is the most important and commonly used prognosis evaluation system [3, 27, 28]. In addition, histological features concerning prognosis should also be taken into consideration. Different pathological subtypes of RCC indicate diverse clinical outcomes [5]. Moreover, the difference in cell differentiation also results in different clinical outcomes [27, 29, 30]. Additionally, the appearance in cellular levels of RCC shows relationship with the prognosis of RCC patients [31]. The International Society of Urological Pathology (ISUP) grading system is a prognostic classification regarding the morphology of histological nuclear abnormality [2, 32, 33]. High ISUP scores indicate advanced nuclear abnormality and unfavorable prognosis [5, 32]. Clinicians make treatment decisions for RCC patients usually based on TNM classification, histological subtypes and ISUP grading [2].

With the development of artificial intelligence, deep learning techniques have been intended to be applied in the analysis of pathological images [2]. Initially researchers mainly targeted in single cell level on immunochemical images [34]. As the rapid progress in deep learning, the multicolored HE-stained whole-slide images were analyzed for various purposes, mainly for precise diagnosis, cancer prognosis prediction and drug resistance evaluation. However, limited studies were targeted on lymph node involvement prediction based on WSIs. Wang et al. evaluated the lymph node involvement of gastric cancer from lymph node WSIs [17]. Wessels et al. applied convolutional neural network to predict the lymph node involvement in prostate cancer WSIs [15]. Moreover, Brockmoeller et al. predicted the lymph node involvement in early-stage colorectal carcinoma patients WSIs through deep learning [16]. In this study, we judged the lymph node involvement of RCC through the origin RCC histological images by deep learning and verified the performance of the model in frozen-pathological section WSIs.

Lymph node involvement is a critical event for advanced RCC patients [35]. The appearance of lymph node involvement and remote metastasis represent for late stages of RCC, which usually show poor clinical outcomes and unfavored drug responses [7, 36]. Currently, the evaluation of lymph node involvement is mainly through computed tomography, magnetic resonance imaging and ^18^F-fluorodeoxyglucose positron emission tomography/computed tomography [7]. The golden standard of lymph node involvement assessment is histopathological confirmation based on lymph node dissection. However, it remains unclear that the role of lymph node dissection during radical RCC surgery [12]. It is estimated that only 14.8% RCC patients underwent lymph node dissection are confirmed pathological lymph node involvement [37]. The American Urological Association suggests to take lymph node dissection for RCC patients with suspicious regional lymph node involvement based on computed tomography, magnetic resonance imaging and ^18^F-fluorodeoxyglucose positron emission tomography/computed tomography images [12, 37]. However, there is a huge gap between clinical lymph node positive and pathological lymph node positive [13]. Furthermore, the evaluation of micrometastasis is difficult from radiological imaging [38]. Hence, an improved parameter for lymph node dissection is necessary.

Unfortunately, even pathologists still could neglect some micrometastasis in lymph nodes [14]. Considering the workload, pathological technicians usually resect one HE-slide for each lymph node sample. Nevertheless, some micrometastasis occupy very limited space of a lymph node and can be detected only after serial Sect. [14]. In addition, RCC patients with micrometastasis in lymph nodes still show poor prognosis [13]. Therefore, our D_LNM_ could be a meaningful indicator for pathologists to apply serial sections on micrometastasis diagnosis.

According to pathological diagnosis guideline, the invasion of micro-lymphatic vessels in RCC should be mentioned in the pathological diagnosis reports [5]. Tumor cells initially invade mirco-lymphatic vessels in the kidney, transport through peripheral lymphatic vessels and eventually erode a whole lymph node [17]. As a result, the invasion of micro-lymphatic vessels can be regarded as the pre-lymph node involvement and indicated for unfavorable prognosis [39, 40]. Shoup et al. found that the presence of lymphovascular invasion showed correlation with lymph node involvement [41].

The atypical nuclear appearance, including nuclear pleomorphism, abnormal nucleus- to-cytoplasm ratio, enlarged nucleoli and pathological mitosis, is a poor prognostic indicator [41–44]. The nuclear pleomorphism stands for variance in nuclear shape and morphology [43]. Studies reveal the correlation between nuclear pleomorphism and lymph node involvement [41, 43]. In addition, Conversano et al. found that the amount of mitosis, especially pathological mitosis, was related to lymph node involvement [44].

There are some limitations in this study. Firstly, the model needs to be further evaluated among RCC patients from different hospitals and regions. Secondly, the model was based on retrospective studies. As a result, the model needs further prospective validation. Thirdly, it is also important to integrate the molecular signatures of RCC to improve the prediction accuracy of the model.

## Conclusions

In conclusion, we developed and verified a deep learning-based strategy for predicting lymphatic metastasis from primary RCC WSIs, which could be applied in frozen-pathological sections and act as a prognostic factor for RCC to distinguished patients with worse survival outcomes. However, further validation of our D_LNM_ in prospective patient cohorts should also be performed for clinical practices.

### Electronic supplementary material

Below is the link to the electronic supplementary material.

Supplementary Material 1

## Data Availability

All data generated or analyzed during this study are included in this published article.

## Code availability

All code related to this method was written in Python. Custom code related to the deep learning models is available at https://github.com/mahmoodlab/CLAM.
