# Supplementary material for: Deep learning-based pathological prediction of lymph node metastasis for patient with renal cell carcinoma from primary whole slide images
Source: J Transl Med. 2024 Jun 14;22:568. doi: 10.1186/s12967-024-05382-6 (PMC11177484; doi:10.1186/s12967-024-05382-6)
Supplement: Supplementary file 1 — Supplementary Material 1 [file 12967_2024_5382_MOESM1_ESM.docx]

**Table S1**. Basic clinical characteristics of patients from three independent patient cohorts.

|  | **SGH Cohort (402)** |  | **TCGA Cohort (381)** |  | **CPTAC Cohort (112)** |
| --- | --- | --- | --- | --- | --- |
| **Age(years)** |  |  |  |  |  |
| ≥65 | 140(34.8%) |  | 146(38.3%) |  | 33(29.5%) |
| ＜65 | 262(65.2%) |  | 235(61.7%) |  | 79(70.5%) |
| **Sex** |  |  |  |  |  |
| Male | 289(71.9%) |  | 585(66.0%) |  | 79(70.5%) |
| Female | 113(28.1%) |  | 302(34.0%) |  | 33(29.5%) |
| **Stage** |  |  |  |  |  |
| i | 364(90.6%) |  | 143(37.5%) |  | 38(33.9%) |
| ii | 25(6.2%) |  | 60(15.7%) |  | 12(10.7%) |
| iii | 13(3.2%) |  | 109(28.6%) |  | 36(32.1%) |
| iv | 0 |  | 60(15.7%) |  | 26(23.2%) |
| Unknown | / |  | 9(2.3%) |  | / |
| **Grade** |  |  |  |  |  |
| G1 | 47(11.7%) |  | 3(0.8%) |  | 0 |
| G2 | 204(50.8%) |  | 103(27.0%) |  | 60(53.6%) |
| G3 | 50(12.4%) |  | 102(26.8%) |  | 40(35.7%) |
| G4 | 6(1.5%) |  | 36(9.4%) |  | 12(10.7%) |
| Unknown | 95(23.6%) |  | 137(36.0%) |  | / |
| **T stage** |  |  |  |  |  |
| T1 | 365(90.8%) |  | 155(40.7%) |  | 38(33.9%) |
| T2 | 28(7.0%) |  | 72(18.9%) |  | 14(12.5%) |
| T3 | 9(2.2%) |  | 145(38.0%) |  | 54(48.2%) |
| T4 | 0 |  | 9(2.4%) |  | 6(5.4%) |
| **N stage** |  |  |  |  |  |
| N1 | 5(1.2%) |  | 50(13.1%) |  | 10(8.9%) |
| N0 | 397(98.8%) |  | 331(86.9%) |  | 102(91.1%) |
| **M stage** |  |  |  |  |  |
| M1 | 0 |  | 13(3.4%) |  | 24(21.4%) |
| M0 | 402(100%) |  | 84(22.1%) |  | 71(63.4%) |
| Unknown | / |  | 284(74.5%) |  | 17(15.2%) |
| **Subtype** |  |  |  |  |  |
| ccRCC | 307(76.4%) |  | 249(65.4%) |  | 112(100%) |
| pRCC | 51(12.7%) |  | 76(19.9%) |  | 0 |
| ChRCC | 44(10.9%) |  | 56(14.7%) |  | 0 |
| **Survival status** |  |  |  |  |  |
| Dead | 13(3.2%) |  | 128(33.6%) |  | 15(13.4%) |
| Alive | 294(73.1%) |  | 251(65.9%) |  | 97(86.6%) |
| Unknown | 95(23.6%) |  | 2(0.5%) |  | / |

TCGA, the Cancer Genome Atlas; Shanghai General Hospital, SGH; CPTAC, Clinical Proteomic Tumor Analysis Consortium; ccRCC, clear cell renal cell carcinoma; pRCC, papillary renal cell carcinoma; ChRCC, chromophobe renal cell carcinoma.
